# A CARD9 Polymorphism Is Associated with Decreased Likelihood of Persistent Conjugated Hyperbilirubinemia in Intestinal Failure

**DOI:** 10.1371/journal.pone.0085915

**Published:** 2014-01-21

**Authors:** Karolina Maria Burghardt, Vishal Avinashi, Christina Kosar, Wei Xu, Paul W. Wales, Yaron Avitzur, Aleixo Muise

**Affiliations:** 1 Group for Improvement of Intestinal Function and Treatment (GIFT), Transplant Centre, Toronto, Ontario, Canada; 2 Division of Gastroenterology, Hepatology and Nutrition, Department of Pediatrics, Toronto, Ontario, Canada; 3 SickKids IBD Centre, Program in Cell Biology, Hospital for Sick Children, University of Toronto, Toronto, Ontario, Canada; 4 Division of Gastroenterology, Hepatology and Nutrition, Department of Pediatrics, BC Children Hospital, Vancouver, British Columbia, Canada; 5 Dalla Lana School of Public Health, University of Toronto, Toronto, Ontario, Canada; Westfaelische Wilhelms Universitaet, Germany

## Abstract

Recently, genetic associations have been described in intestinal transplants. Namely, Crohn's disease susceptibility gene NOD2 polymorphisms have been reported to be more prevalent in patients with graft failure following intestinal transplantation (IT). Therefore, we sought to determine if polymorphisms in the NOD2 signaling cascade, including NOD2, CARD9, RAC1 and ATG16L1 are associated with intestinal failure (IF) or its complications. We carried out a cross-sectional study of 59 children with IF and 500 healthy Caucasian controls. Using the Taqman platform we determined the prevalence of NOD2 as well as ATG16L1, RAC1 and CARD9 SNPs. NOD2 pathway polymorphisms were evaluated in relation to outcomes of episodes of sepsis, ICU admissions, hyperbilirubinemia and need for IT. We found that the minor allele of a *CARD9* SNP was associated with protection from developing IF when compared to healthy controls and was also associated with decreased odds of sustained conjugated hyperbilirubinemia. Therefore, IF patients with CARD9 polymorphism are less likely to develop progressive liver disease and suggests that host innate immunity may play a role in IF associated liver disease.

## Introduction

Intestinal failure (IF) is defined by the anatomic or functional loss of digestive and absorptive functions as a result of a short bowel syndrome (ex. volvulus, NEC, gastroschisis), motility disorders or other congenital intestinal conditions (ie. tufting enteropathy and microvillus inclusion disease). Although the group consists of heterogeneous etiologies, the commonality is their progression to similar complications, namely bacterial overgrowth, recurrent line infections, thrombosis of central veins and liver disease. Intestinal failure associated liver disease (IFALD) is among the major determinants of morbidity and mortality in children with IF.[1] The success of treating these patients relies on vigilant early medical intervention. There are currently no biochemical or clinical predictors for the likelihood of these complications in this vulnerable patient population. Grouping individuals according to their genetic susceptibility to adverse outcomes has the potential of tailoring follow up and medical interventions based on the patient's genotype and risk category.

In fact, the study of genomics and stem cell biology in medicine is increasingly directed toward individualized care for subpopulations of patients with specific genetic disease pathology, phenotype or disease complications in an effort to understand the pathophysiology and potentially offer diagnostic as well as therapeutic tools earlier in the disease evolution.[2], [3] Among other examples are studies related to NOD2 (nucleotide-binding oligomerization domain-containing protein 2), an intracellular microbial sensor that plays a role in intestinal immune homeostasis. Patients with Crohn's disease having specific polymorphisms in NOD2 are more likely to have an earlier onset of disease, ileal involvement, fibrostenosis and an increased likelihood for ileocecal surgery.[4] In IF patients, the NOD2 polymorphism rs2066844 was more frequent in patients with short gut syndrome that required a combined liver-intestine transplant [5] and in those patients who had graft rejection post intestine transplantation [6]. However, the molecular signaling pathways related to the NOD2 gene defect are complex and its role in the pathogenesis of inflammatory diseases is still unclear.

Among the NOD2 downstream signaling molecules most closely linked to intestinal inflammation and inflammatory bowel disease are caspase recruitment domain-containing protein (CARD)9, RAC1 and ATG16L1. Specifically, CARD9 is a non-redundant adaptor molecule that stimulates T cells to differentiate into TH17 cells and mediates innate immunity as well as inflammation.[7] ATG16L1 is involved in signaling of bacterial-targeted autophagosomes.[8] And, RAC1 is a molecule that influences Toll-Like-Receptor-2 (TLR2) regulation, leukocyte chemotaxis, barrier defense and bacterial killing. [9] One hypothesis proposes that the presence of impaired NOD2 sensors or their downstream signaling molecules results in an altered innate immunity and gut homeostasis. We sought to determine if polymorphisms of NOD2 genes or their downstream signaling molecules are associated with IF and with different clinical outcomes in patients with intestinal failure (IF). This is the first report to show CARD 9 polymorphism is of particular significance to patients with IF.

## Methods

### Patient recruitment and ethics

The study is a cross-sectional genetic study of living patients with a known diagnosis of intestinal failure whose medical care is managed by the intestinal rehabilitation program (Group for Improvement of Intestinal Function and Treatment, GIFT) at the Hospital for Sick Children. Written informed consent from the next of kin, caretakers, or guardians on the behalf of the minors/children participants involved in the study was obtained from all participants. Ethics approval for this study including the written informed consent was obtained from the Hospital for Sick Children Research Ethics Board. A total of 59 participants who were followed by our program for 1 year or more were recruited from the GIFT clinic between September 1^st^, 2010 and Dec 31^st^, 2011. In each case, consent was obtained for a one-time blood sampling and review of patient's medical file, in accordance with the SickKids Research Ethics Board guidelines. Written informed consent was obtained from all participants. All outcome clinical data were collected till the end of the study or the date of transplant, which ever came first. Healthy controls were obtained from the Centre for Applied Genomics (Ontario Population Genomics Platform (plates used: 1–5; a complete description of this control population can be found at http://www.tcag.ca/cyto_population_control_DNA.html.

### Study Definitions

Intestinal failure: defined as a loss of more than 75% of small bowel length expected for gestational age or a dependence on total parenteral nutrition (TPN) for more than 42 days.[10]

Bacterial sepsis: defined as blood culture or central venous catheter line culture positive for bacteria. We tallied the number of episodes in the first year of parenteral nutrition use.

Fungal sepsis: defined as blood culture or central venous catheter line culture positive for fungi. We tallied the total number of documented fungal episodes.

ICU admission: defined as admission to the intensive care unit that was not related to peri-surgical recovery or part of the initial neonatal NICU period.

Conjugated hyperbilirubinemia: defined as a sustained 2-week period of conjugated bilirubin level of more than 100 micromol/L, which was not part of an episode of sepsis, urinary tract infection or peri-operative period.

### Genetic analysis

Genotyping was carried out at the SickKids Centre for Applied Genomics (TCAG). Using Taqman platform we determined the prevalence of NOD2 SNPs (rs2066844, rs2066845, rs2066847) and that of its signaling molecules: ATG16L1 (rs2241880), RAC1 (rs10951982), and CARD9 (rs4077515) as we have done previously.[9], [11], [12], [13], [14] Comparisons in the frequency of the minor allele were made between 59 pediatric cases and 500 healthy Caucasian adult controls as described above. NOD2 pathway polymorphisms were evaluated in relation to the following IF related complications: total number of episodes of sepsis, ICU admissions, liver failure (defined as sustained conjugated hyperbilirubinemia of ≥100 micromol/L) and need for intestine or liver transplant.

### Statistics

Descriptive statistics were reported as median and range for continuous variables and frequencies and proportions for categorical variables. PLINK version 1.06 was used to obtain descriptive statistics of the SNPs such as the allele frequency and genotype distribution, it was also used to test for Hardy-Weinberg equilibrium (HWE) for each marker based on Pearson's chi-square test.[15] Although we used a dominant genetic model for primary analysis,[16] we also explored additive and recessive genetic models for sensitivity analysis. Throughout this report the p-values are the dominant genetic model. Both univariate and multivariate analysis have been applied for genetic association analysis. The multivariate model was constructed based on backward selection algorithm. The candidate clinical factors were ≥2 episodes of sepsis, ≥1 episode of fungal sepsis, ≥1 ICU admission, conjugated bilirubin ≥50 micromol/L, conjugated bilirubin ≥100 micromol/L, TPN duration of ≥1 year, listed for transplant, or status post transplanted. Odds ratios (OR) and 95% confidence intervals (CI) were estimated. Two-sided statistical tests were applied. The analyses are performed with SAS 9.2 (SAS Institute, Cary, NC, USA).

## Results

### Demographics

The 59 children recruited to our study tended to born prematurely (n = 37, 65%; median gestational age of 35 weeks) with the majority having an anterior wall defect (n = 14, 24%), NEC (n = 12, 21%) or intestinal atresia (n = 12, 21%) as the etiology of their IF (Table 1). The median birth weight was 2323 g. The majority of patients were Caucasian (n = 26, 44%), Asian (n = 14, 24%) or mixed ethnicity (n = 10; 17%; Table 1).

**Table 1:** Clinical characteristics of study population.

**Clinical parameters**	
Median birth weight (range 630–4120 grams)	2323 g
Median age (range 0.7 – 18.1 years)	4.1 years
Median gestational age (range 26–40 weeks)	35 weeks
Median length of parenteral nutrition (range 10–3848 days)	366 days
	n (%)
Early premature infants (<33 weeks)	13 (22%)
Late premature infants (33–37 weeks)	25 (43%)
Term infants (>37 weeks)	20 (34%)
Gender - males	32 (54%)
**Ethnicity**	
Caucasian	26 (44%)
Asian	14 (24%)
Mixed race	10 (17%)
African	4 (7%)
First Nations	2 (3%)
Middle Eastern	2 (3%)
Hispanic	1 (2%)
**Etiology**	
Anterior wall defect	14 (24%)
Intestinal atresia	13 (22%)
Necrotizing enterocolitis	12 (20%)
Hirschsprung's disease	7 (12%)
Volvulus	3 (5%)
Other	4 (7%)
Microvillous inclusion disease	3 (5%)
Tufting enteropathy	2 (3%)
Chronic diarrhea (not yet diagnosed)	1 (2%)

### Clinical Outcomes

Among the 59 patients with intestinal failure, 30 patients (51%) had ≥2 episodes of sepsis in the first year of TPN use and 20% (n = 11) had ≥1 episode of fungal sepsis during this time (Table 2). Although half of these patients (n = 31) were receiving parenteral nutrition for more than one year, only a quarter of them (n = 15) had conjugated hyperbilirubinemia of ≥100 micromol/L. Only 10% required ICU admission after they were discharged home from the hospital. Transplant assessment was initiated in 20% (n = 12) and 11 were listed for either isolated liver or small bowel or combined liver and small bowel. Out of the 11 patients listed for transplant, 6 patients received a transplant organ, one remained active on the transplant list at the end of the study and 4 were de-listed because of clinical improvement. Of the 6 who received a transplant organ, 3 children received an isolated liver transplant (50%), 2 received a liver and small bowel transplant (33%) and 1 received an isolated small bowel transplant (17%). All children were alive at the end of the study.

**Table 2:** Frequency of clinical outcomes.

Clinical outcome parameters	Number of positive patients
≥2 episodes of sepsis	30 (50.8%)
≥1 fungal infection episode	12 (20.3%)
≥1 ICU admission	6 (10.2%)
Conjugated bilirubin ≥50 micromol/L	25 (42.4%)
Conjugated bilirubin ≥100 micromol/L	15 (25.4%)
TPN >1 year	31 (52.5%)
**Transplant related parameters**	
Assessment for transplant	12 (20.3%)
Listed for transplant	11 (18.6%)
**Patients who underwent transplant**	6 (10.2%)
Isolated liver transplant	3 (5.1%)
Isolated small bowel transplant	1 (1.7%)
Liver and small bowel transplant	2 (3.4%)
**Patients active on transplant list**	1 (1.7%)
**Patients de-listed given improved clinical status**	4 (6.8%)

### Genetic analysis

We compared our cohort of 59 pediatric cases with IF to 500 healthy controls and found that the frequency of the minor allele of CARD9 was more prevalent in healthy controls than in children with intestinal failure (OR of 0.55, 95% CI: 0.32–0.94; p = 0.03). However, there were no differences in the prevalence of the minor allele for the three genetic polymorphisms of NOD2 associated with Crohn's disease (SNPs: rs2066844, rs2066845, rs2066847) as previously reported.[17] There was also no difference in the frequency of RAC1 or ATG16L1 polymorphism between the two groups. (Table 3).

**Table 3:** Genetic analysis of the frequency of minor allele of NOD2, CARD9, RAC1 and ATG16L1 polymorphisms in patients and controls.

Genes	Chromosome	SNP	Minor allele	Allele Frequency		OR	95% CI		P
				Cases	Controls				
ATG16L1	2	rs2241880	G	0.49	0.49	0.9	0.49	1.65	0.72
RAC1	7	rs10951982	A	0.24	0.26	0.87	0.5	1.49	0.61
**CARD9**	**9**	**rs4077515**	**T**	**0.34**	**0.40**	**0.55**	**0.32**	**0.94**	**0.03**
NOD2	16	rs2066844	T	0.01	0.05	0.17	0.02	1.25	0.08
NOD2	16	rs2066845	C	0.02	0.02	1.17	0.26	5.28	0.84
NOD2	16	rs2066847	I	0.02	0.02	0.69	0.16	3.03	0.63

We next examined whether there were any differences in the frequency of the polymorphisms of NOD2, CARD9, RAC1 and ATG16L1 in relation to major clinical outcomes (conjugated hyperbilirubinemia, sepsis episodes, use of parenteral nutrition for >1 year and need for transplantation) among the 59 children with IF. Patients carrying the minor allele of CARD9 had a decreased odds of sustained conjugated hyperbilirubinemia of ≥100 micromol/L (p = 0.04; OR 0.25, 95% CI: 0.07–0.91; Table 4) and thus a lower likelihood of progressive liver disease. The logistic regression analysis showed that the presence of the minor allele was statistically a dominant effect, with a single minor allele needed to confer lower odds of hyperbilirubinemia. We observed a similar trend when considering sustained conjugated hyperbilirubinemia of ≥50 micromol/L (p = 0.05, OR 0.35, 95% CI: 0.12–1.01, Table 5). In addition, we observed that patients having the minor allele of ATG16L1 tended to have decreased odds of more than 2 episodes of sepsis (p = 0.05, OR 0.48, 95% CI: 0.23–1.00); however, this observation was not statistically significant. RAC1 and NOD2 polymorphism was not associated with any of the clinical outcomes. The results of the multivariate analysis are consistent to the univariate analysis.

**Table 4:** Genetic analysis of the frequency of minor allele of NOD2, CARD9, RAC1 and ATG16L1 polymorphisms in IF patients with conjugated hyperbilirubinemia (CB≥100 µmol/L).

Genes	Chromosome	SNP	Minor Allele	Allele Frequency		OR	95% CI		P	P_adj*
				CB≥100	CB<100					
ATG16L1	2	rs2241880	G	0.53	0.48	3.03	0.60	15.3	0.18	0.34
RAC1	7	rs10951982	A	0.23	0.24	0.88	0.27	2.91	0.83	0.98
**CARD9**	**9**	**rs4077515**	**T**	**0.17**	**0.40**	**0.25**	**0.07**	**0.91**	**0.04**	**0.03**
NOD2	16	rs2066844	T	0	0.01	N/A	N/A	N/A	1	1
NOD2	16	rs2066845	C	0.07	0	N/A	N/A	N/A	1	1
NOD2	16	rs2066847	I	0	0.02	N/A	N/A	N/A	1	1

* : P_adj are the genetic association analysis p-values from the multivariate analysis.

**Table 5:** Genetic analysis of the frequency of minor allele of NOD2, CARD9, RAC1 and ATG16L1 polymorphisms in IF patients with conjugated hyperbilirubinemia (CB≥50 µmol/L).

Genes	Chromosome	SNP	Minor Allele	Allele Frequency		OR	95% CI		P	P_adj*
				CB≥50	CB<50					
ATG16L1	2	rs2241880	G	0.52	0.47	1.91	0.57	6.44	0.30	0.08
RAC1	7	rs10951982	A	0.22	0.25	0.84	0.30	2.42	0.75	0.55
**CARD9**	**9**	**rs4077515**	**T**	**0.24**	**0.41**	**0.35**	**0.12**	**1.01**	**0.05**	**0.05**
NOD2	16	rs2066844	T	0.02	0	N/A	N/A	N/A	1	1
NOD2	16	rs2066845	C	0.04	0	N/A	N/A	N/A	1	1
NOD2	16	rs2066847	I	0.04	0	N/A	N/A	N/A	1	1

* : P_adj are the genetic association analysis p-values from the multivariate analysis.

Lastly, we examined whether there were any differences in the frequency of the polymorphisms of NOD2, CARD9, RAC1 and ATG16L1 in relation to inflammatory etiologies of IF such as in the cases with necrotizing enterocolitis (NEC). None of the polymorphisms queried were associated with NEC in our patient cohort (data not shown).

## Discussion

Patients with intestinal failure associated liver disease have a much lower rate of survival than their non-cholestatic counterparts. In a cohort of 78 patients with short bowel syndrome who had conjugated hyperbilirubinemia (>34 micromol/L), the rate of survival was 20% versus 80% in those with no cholestasis.[18] The pathogenesis of IFALD is complex, multifactorial and poorly understood. Among the contributors are bacterial endotoxins, components of parenteral nutrition and activation of the innate immune system. The liver is strategically positioned to mediate immune tolerance versus inflammation since it is continually exposed to antigens from ingested food and bacteria in the gastrointestinal tract. Liver cells express an array of Toll-like receptors (TLR1-9), which are a family of receptors that recognize specific pathogen-associated molecular patterns (PAMP) such as those of lipopolysaccharide (LPS).[19] A variety of TLRs are hypothesized to be key players in the gut-liver axis that mediate innate inflammatory responses and subsequent organ-injury. For example, in a mouse model that mimicked chronic parenteral nutrition use and intestinal injury, liver inflammation was correlated with higher levels of LPS in the portal vein and increased transcription of pro-inflammatory cytokines (IL6, TNF-alpha and TGF-beta) in Kupffer cells. [20] Interestingly, abrogation of the LPS-TLR-4 signaling in these mice was associated with a decreased transcription of IL-6 in Kupffer cells and markedly attenuated liver injury. Furthermore, animal studies found that genetically susceptible IL-10 -/- knockout mice are protected against chronic intestinal inflammation in the absence of TLR-4 but develop colitis in the absence of TLR-2.[21] Therefore, these findings highlight the importance of TLRs and their role in mediating progressive liver disease and intestinal inflammation. Given that the signaling pathway for TLR-4 involves CARD9 as a central molecular scaffold in mediating inflammatory response, CARD9 may in fact be a key determinant in the evolution of inflammation in the liver and IFALD.

Animal models and molecular studies have reported that the innate immune response to bacterial, viral and fungal stimuli can be mediated via CARD9, which orchestrates varying signaling pathways and polarize T-helper cell reactions.[22], [23] Once these antigenic stimuli are recognized by TLRs or non-TLRs such as dectin-1 or trem-1, the signals converge on key adaptor proteins like CARD9, which modulate the innate immune response to the invading microbial agents. For instance animals whose macrophages and dendritic cells are CARD9 deficient are unable to mount an adequate host defense and succumb to *Candida albicans* infection. [24], [25] The integration of all these immune pathways results in CARD9 being involved in intestinal epithelial cell restitution following colonic injury secondary to infection as well as following DSS chemical, a model of IBD colitis.[26]

A common genetic variation of CARD9 is at amino acid at position 12, resulting in a substitution of a serine for an asparagine (SNP rs4077515). Caucasian and African populations have a high frequency of the minor allele of this polymorphism, 53 and 25%, respectively.[27] In humans, a homozygous mutation and thus loss of function of CARD9 is rare, with a single case report of a family having increased susceptibility to fungal infection in association with the CARD9 deficiency.[28] Heterozygous inheritance of the minor allele likely leads to a change of function of the CARD9 signaling pathway. In some, inheritance of the minor allele single nucleotide polymorphisms, SNP rs4077515, confers susceptibility to Crohn's or ulcerative colitis (GWAS analysis with Odds ratio of 1.2 in both diseases).[29], [30], [31] In addition, the CARD9 SNP rs4077515 was also associated with primary sclerosing cholangitis in patients with UC.[32] However, interestingly a rare variant of this CARD9 SNP disrupts exon splicing, resulting in reduce levels of functioning CARD9 and was found to be protective to the development of IBD.[33] These findings highlight the importance of CARD9 polymorphisms and its influence on the evolution of inflammatory diseases.

In our study, we found that the frequency of the minor allele of CARD9 was higher in healthy adults than in children with intestinal failure (OR of 0.55; p = 0.029). Congruent with this finding we observed that among the patients with intestinal failure, those having the CARD9 polymorphism (SNP rs4077515) had a lower likelihood of progressive liver disease. This suggests that the response of the innate immune system to bacterial and food antigens is different as a result of this CARD9 variant. One possibility is a different signaling cascade through TLR and the CARD9 polymorphism that may skew the immune system away from an inflammatory response in the liver. Perhaps the infiltration by macrophages into the intestine and/or liver is decreased in these individuals, similar to the findings in an animal model of carditis, where CARD9 deficient mice showed suppressed macrophage infiltration and cytokine expression in the cardiac muscle.[34] The molecular pathways to support these hypotheses remain to be elucidated.

Our analysis of the NOD2, ATG16L1 and RAC1 polymorphisms was not significantly associated with progressive liver disease or frequent episodes of sepsis, ICU admissions, chronic TPN use and need for transplantation. We did observe a trend suggestive of a lower likelihood of bacterial sepsis (OR 0.47, 95% CI: 0.04-5.26, p = 0.54) in patients with the minor allele of ATG16L1 (rs2241880). The significance of this finding is unclear but it again highlights the potential importance of the innate immune signaling molecules in the evolution of IF related complications. The ATG16L1 interacts with NOD2 in a signaling cascade that leads to autophagy of bacterial fragments and antigen presentation by dendritic cells.[8] We also did not observe a higher frequency of the high risk NOD2 alleles reported in Crohn's disease (SNPs: rs2066844, rs2066845, rs2066847) in our population as compared to healthy adult controls and as risk alleles for IF complications. This is due to the rarity of these SNPs in the general population.

One study performed genetic analysis of adult IT recipients over a three-year period and reported that 35% of their patients had the high risk NOD2 alleles reported in Crohn's disease, and these were also associated with a 100 fold higher likelihood of allograft failure.[17] Subsequent studies of largely pediatric patients could not replicate the increased likelihood of allograft rejection or loss. [5], [35] The incongruity in the reported findings remains unclear. Among the explanations may be that adult patients most often receive isolated small bowel transplants while multi-visceral transplants are more common in pediatric patients due to different etiologies of IF. Overall, these studies emphasize the recurrent limitations in teasing out the genetic contributors to clinical outcomes in IF, which include relatively small sample size, heterogeneity of etiologies and variability of transplant type.

Our study also has several of these limitations. First, the small sample size limits genetic statistical analysis. Therefore, the finding of statistical differences is most notable in this exploratory and hypothesis generating study. It is important to note the lesser prevalence of IF in relation to other diseases such as IBD, PSC or ankylosing spondylitis, where CARD9 has been shown to be relevant to the underlying inflammatory processes.[7] Therefore, our study highlights the need for larger multi-center studies to validate the results and further investigate the pathophysiology behind these observations. In addition, our study is hypothesis driven and examined a restricted and focused series of gene polymorphisms that were selected *a priori* based on our review of the current literature and the assumption that Crohn's related genes play a role in IF. It is possible that a GWAS approach on a large population of patients will reveal additional genes that influence the outcome of children with IF. Deep sequencing is one example of the success of such approaches in elucidating the IL-10 gene mutations associated with another low-prevalence disease subset, very early onset IBD. [36]

## Conclusion

Genetic alterations in host innate immunity, specifically to CARD9 may be associated with varying susceptibility to IF as well as to IFALD. Further studies are needed to elaborate the signaling pathways and modulators of the innate immune system that promote progressive liver disease in patients with IF.
